# Effect of C reactive protein point-of-care testing on antibiotic prescribing for lower respiratory tract infections in nursing home residents: cluster randomised controlled trial

**DOI:** 10.1136/bmj.n2198

**Published:** 2021-09-21

**Authors:** Tjarda M Boere, Laura W van Buul, Rogier M Hopstaken, Maurits W van Tulder, Jos W M R Twisk, Theo J M Verheij, Cees M P M Hertogh

**Affiliations:** 1 Department of Medicine for Older People, Amsterdam Public Health Research Institute, Amsterdam UMC, VU University Medical Center, Amsterdam, Netherlands; 2 Primary Health Care Center, Hapert en Hoogeloon, Hapert, Netherlands; 3 Star-shl Diagnostic Centers, Etten-Leur, Netherlands; 4 Department of General Practice, CAPHRI School for Public Health and Primary Care, Maastricht University Medical Centre, Maastricht, Netherlands; 5 Faculty of Behavioural and Movement Sciences, Amsterdam Movement Sciences Research Institute, Vrije Universiteit Amsterdam, Amsterdam, Netherlands; 6 Department of Epidemiology and Biostatistics, Amsterdam UMC, VU University Medical Center, Amsterdam, Netherlands; 7 National Institute for Public Health and the Environment (RIVM), Bilthoven, Netherlands; 8 Julius Center for Health Sciences and Primary Care, University Medical Center Utrecht, Utrecht, Netherlands

## Abstract

**Objective:**

To evaluate whether C reactive protein point-of-care testing (CRP POCT) safely reduces antibiotic prescribing for lower respiratory tract infections in nursing home residents.

**Design:**

Pragmatic, cluster randomised controlled trial.

**Setting:**

The UPCARE study included 11 nursing home organisations in the Netherlands.

**Participants:**

84 physicians from 11 nursing home organisations included 241 participants with suspected lower respiratory tract infections from September 2018 to the end of March 2020.

**Interventions:**

Nursing homes allocated to the intervention group had access to CRP POCT. The control group provided usual care without CRP POCT for patients with suspected lower respiratory tract infections.

**Main outcome measures:**

The primary outcome measure was antibiotic prescribing at initial consultation. Secondary outcome measures were full recovery at three weeks, changes in antibiotic management and additional diagnostics during follow-up at one week and three weeks, and hospital admission and all cause mortality at any point (initial consultation, one week, or three weeks).

**Results:**

Antibiotics were prescribed at initial consultation for 84 (53.5%) patients in the intervention group and 65 (82.3%) in the control group. Patients in the intervention group had 4.93 higher odds (95% confidence interval 1.91 to 12.73) of not being prescribed antibiotics at initial consultation compared with the control group, irrespective of treating physician and baseline characteristics. The between group difference in antibiotic prescribing at any point from initial consultation to follow-up was 23.6%. Differences in secondary outcomes between the intervention and control groups were 4.4% in full recovery rates at three weeks (86.4% *v* 90.8%), 2.2% in all cause mortality rates (3.5% *v* 1.3%), and 0.7% in hospital admission rates (7.2% *v* 6.5%). The odds of full recovery at three weeks, and the odds of mortality and hospital admission at any point did not significantly differ between groups.

**Conclusions:**

CRP POCT for suspected lower respiratory tract infection safely reduced antibiotic prescribing compared with usual care in nursing home residents. The findings suggest that implementing CRP POCT in nursing homes might contribute to reduced antibiotic use in this setting and help to combat antibiotic resistance.

**Trial registration:**

Netherlands Trial Register NL5054

## Introduction

Antimicrobial resistance is a growing worldwide public health threat. An important driving force is inappropriate antibiotic use.^1^ ^2^ Antibiotic prescription rates are relatively high in the nursing home setting because of high infection burden, frailty, and higher risk of serious clinical outcomes in this population.^3^ ^4^ ^5^ ^6^ For more serious lower respiratory tract infections, such as pneumonia, this risk could increase if appropriate treatment is delayed.^7^ ^8^ However, differentiating serious from less serious lower respiratory tract infections such as acute bronchitis is often difficult. In the nursing home setting this difficulty arises from less distinctive clinical presentations and limited diagnostic resource availability (eg, chest radiograph) or applicability (eg, sputum culture).^3^ ^4^ ^8^ ^9^ ^10^ This diagnostic uncertainty reinforces the choice for antibiotic prescribing to be better safe than sorry, and probably leads to overprescription.^8^ ^11^ ^12^ ^13^ ^14^

C reactive protein point-of-care testing (CRP POCT) for suspected lower respiratory tract infections might contribute to prompt and appropriate decisions of whether or not to prescribe antibiotics, or to suggest additional investigations. CRP is an acute phase protein synthesised by the liver as a non-specific response to inflammatory stimuli. CRP levels respond dynamically to the presence (increase within six hours) and relief (half life of 19 hours) of inflammation.^15^ ^16^ Given the non-specificity of the CRP response, physicians cannot unequivocally differentiate between viral and bacterial causes of infection. However, when a clinical suspicion of lower respiratory tract infection exists, the CRP level does support the physician in assessing the likelihood of serious or self-limiting lower respiratory tract infections.^17^ ^18^ ^19^ ^20^ Procalcitonin, another inflammatory biomarker, is often proposed for diagnostic or prognostic purposes in infections such as sepsis and lower respiratory tract infections, however the evidence remains conflicting.^20^ ^21^ ^22^ ^23^ ^24^ ^25^ Primary care studies have shown that CRP adds diagnostic value to the evaluation of clinical signs and symptoms when predicting bacterial lower respiratory tract infections or pneumonia, whereas procalcitonin does not.^20^ ^26^

Further evidence from primary care has established that CRP POCT is a cost effective tool for reducing antibiotic prescribing for lower respiratory tract infections without negative consequences for clinical recovery^18^ ^27^ ^28^ ^29^; however, in the nursing home setting this is yet to be established. Dutch nursing homes use specialised elderly care physicians who have their principal site of practice within the nursing home,^30^ which could allow centralised and around the clock availability of CRP POCT in this setting. In a cluster randomised controlled trial we evaluated whether CRP POCT results in a safe reduction in antibiotic prescribing for nursing home residents with suspected lower respiratory tract infection compared with usual care.

## Methods

### Trial design and participating centres

We performed a pragmatic, open label, cluster randomised controlled trial. The trial was conducted in accordance with a previously published protocol.^31^ The Medical Ethical Committee of the VU University Medical Centre in Amsterdam approved the trial protocol on 28 March 2018 and the participation of all recruitment sites (nursing homes) in the trial. The trial was registered on the Netherlands Trial Register on 29 August 2018 (trial No NL5054).

We recruited 11 nursing homes across the Netherlands that each accommodated 400 residents on average. Physicians in these nursing homes collected data from September 2018 to the end of March 2020. We used a simple randomisation procedure with a 1:1 ratio, which resulted in six intervention group nursing homes that used CRP POCT and five control group nursing homes that provided care as usual. CRP POCT devices (QuikRead go, Aidian, Espoo, Finland) were provided by primary care diagnostic centre Saltro (Unilabs, Utrecht, Netherlands) for the duration of the trial, including the run-in period.

### Patient enrolment

All somatic, psychogeriatric, and short stay (geriatric rehabilitation and short term residential care) nursing home residents received trial information from the researchers shortly before the start of the trial or upon admission of a new resident to the nursing home during the trial. Patients with a suspected lower respiratory tract infection, according to their physician’s assessment, were eligible for participation. This broad inclusion criterion corresponded with the pragmatic nature of the trial (we did not use a strict definition because of the often atypical and varying clinical presentation). Exclusion criteria were current or recent (in the past week) infection or use of antibiotics, or a recorded statement to withhold antibiotic treatment. A two phase informed consent process was used. The first phase allowed all residents to opt out of participation. In the second phase, physicians asked for written informed consent only from patients who were eligible for participation, during or shortly after initial consultation. The physician asked the patient’s representative for consent if the patient definitely did not have decision making capacity. Deferred consent (informed consent requested after the use of CRP POCT) was obtained when the patient was critically ill or the patient’s representative was not available during the initial consultation.

### Trial procedures

Data on clinical status, additional diagnostics, and management decisions were collected for all participants on initial consultation and one week and three weeks later. During each consultation, treating physicians filled out electronic case report forms that were integrated into the nursing home electronic patient record system. These forms were automatically uploaded (in real time) to the secure database portal of the research team.

Physicians employed by Dutch nursing homes have their principal site of practice within the nursing home.^30^ In the intervention group, the decision to use CRP POCT as part of the diagnostic investigation was left to the discretion of the treating physician. Therefore, participant inclusion in the intervention group was irrespective of CRP POCT use. The physicians in the control group agreed to provide care as usual without CRP POCT. All physicians from each participating site could enrol eligible patients.

### CRP POCT intervention

Intervention group physicians received a medical training session from the research team on the correct use of CRP POCT and its interpretation based on the available evidence and the current Dutch guideline recommendations on lower respiratory tract infections.^32^ Handouts and other instruction materials were provided as a reference source and to guide physicians who could not attend the training session and those who were newly employed. Each nursing home selected a group of physicians and nurses to be trained in the use of CRP POCT; experts from the primary care laboratory provided this technical training. The CRP POCT devices were then installed to enable the trained group to immediately start familiarising themselves with CRP POCT in routine practice. During the trial, decisions on the use and interpretation of CRP POCT were informed by the medical training knowledge and guideline recommendations, but remained at the discretion of the physicians.^32^

### Outcome measures

*Main analysis*—the primary outcome measure of the main analysis was antibiotic prescribing at initial consultation (yes or no). Secondary outcome measures were the use of additional diagnostics, including repeated CRP tests at one and three weeks (yes or no at each time point); changes in antibiotic treatment policy at one and three weeks (yes or no at each time point); complications (descriptively presented) and safety indicators—full recovery at three weeks according to the physician (yes=fully recovered, no=not recovered, partly recovered, or deceased), hospital admission at baseline, one week, or three weeks (yes or no), and all cause mortality at baseline, one week, or three weeks (yes or no).

*Secondary analyses*—we also explored the extent to which total antibiotic prescribing in nursing homes was influenced by any potential difference in antibiotic prescribing for lower respiratory tract infections. We collected pseudonymised pharmacy dispensing data from participating nursing homes on all systemic, short term antibiotic prescriptions (ATC code J01) for all indications during the trial period and eight months preceding the trial. Finally, we studied the range of CRP values within which physicians decided to prescribe antibiotics. CRP values were shown continuously and categorised per 20 mg/L up to 100 mg/L, and 100-200 mg/L.

### Statistical analysis

*Main analysis*—in our protocol paper we presented the full sample size calculation that adjusted for an intra-cluster correlation coefficient of 0.06 and resulted in a total of 671 participants.^31^ The main analysis was performed using intention-to-treat analysis; all participants were analysed in the group they were allocated to, regardless of whether they received the intervention or not. We performed a logistic generalised estimating equation analysis with correction for clustering at the physician level (adjusted model) to correct for dependency of observations. To improve the statistical analysis plan presented in the protocol paper,^31^ we also corrected for baseline characteristics to account for any potential post randomisation differences at baseline (final model). Several baseline characteristics were chosen based on their expected clinical relevance to the outcome measure^32^ and with relevance to potential post randomisation differences: age, sex, nursing home ward, severity of disease, presence of tachypnea, one sided abnormal lung sounds at initial consultation, and comorbid conditions (chronic obstructive pulmonary disease, congestive heart failure, dementia, and diabetes). Additionally, a priori antibiotic prescribing for the nursing home the participant lives in (the average number of prescriptions per 1000 resident days per month from January to September 2018) was derived from pharmacy dispensing data to add as a baseline characteristic. Secondary outcome measures were analysed by using the final model structure (recovery at three weeks, mortality at any point, and hospital admission at any point) and longitudinally (changes in antibiotic management and use of additional diagnostics).

*Secondary analyses*—we plotted categories of CRP values against the decision for antibiotic treatment at initial consultation with a stacked diagram. We aimed to explore descriptively at which point—for which range of CRP values—antibiotics were increasingly prescribed. Also, we plotted physician specific antibiotic prescribing decisions across continuous CRP values. For the analysis of pharmacy dispensing data, we calculated the average number of antibiotic prescriptions per 1000 resident days per month and defined daily dose. We plotted the monthly defined daily dose and average number of prescriptions for the intervention and control groups before and during the trial period to explore potential trend differences.

For missing data in the pharmacy dispensing data we imputed the standard dosage, or if this was not possible, we used the average value of other prescriptions for the same drug type. For prescriptions with no chronic or short term description, we excluded prescriptions with treatment durations >70 days. After further inspection of indications, we also excluded prescriptions with treatment durations >42 days.

We used SPSS statistical software package version 26 for descriptive statistics. For the logistic generalised estimating equation analysis, Stata statistical software package version 14 was used.

### Patient and public involvement

No patients were involved in setting the research question or the outcome measures, nor were they involved in developing plans for recruitment, design, or implementation of the study. No patients were asked to advise on interpretation or writing up of results. The nursing home organisations involved their client councils before study commencement, which was either to inform the council about the study participation, or to ask the council for their consent to participate in the study. Some client councils of participating nursing homes reviewed the information letter before study commencement.

## Results

A total of 242 patients from 11 nursing home organisations were included in the UPCARE study. One participant retrospectively did not fit the inclusion criteria. We did not have any baseline data of three other participants. Baseline characteristics were comparable between the trial groups (table 1). Sixty four percent of the study population was female and the average age was 84.4 years (standard deviation 8.2). The most common chronic conditions were congestive heart failure, chronic obstructive pulmonary disorder, and dementia. Most patients were moderately ill (77%) and presented with a cough (73%) at initial consultation. Other frequently presented signs and symptoms were abnormal lung sounds (63% of patients) and dyspnea (60% of patients). CRP POCT was used in 87.4% of patients in the intervention group (table S3) and the median CRP level was 32.5 mg/L (table 1). 

**Table 1:** Study population characteristics and clinical status at initial consultation. Data are numbers (percentages) unless stated otherwise

Characteristics	Total study population* (n=241)	Intervention group* (n=162)	Control group* (n=79)
**Patient characteristics**			
Age (years), mean (SD)	84.4 (8.2)	84.3 (8.1)	84.5 (8.4)
Women	153 (64)	104 (64)	49 (62)
**Nursing home ward**			
Psychogeriatric	78 (33)	55 (35)	23 (29)
Somatic	113 (48)	71 (45)	42 (53)
Geriatric rehabilitation	40 (17)	29 (18)	11 (14)
Short term residential care	6 (3)	3 (2)	3 (4)
**Comorbid diseases**			
Cerebrovascular accident	47 (20)	32 (20)	15 (19)
Congestive heart failure	69 (29)	50 (31)	19 (24)
Chronic obstructive pulmonary disease	76 (32)	47 (30)	29 (37)
Dementia	69 (29)	44 (28)	25 (32)
Diabetes	47 (20)	29 (18)	18 (23)
Kidney failure	5 (2)	3 (2)	2 (3)
**Clinical status at baseline**			
CRP value, median (IQR)	NA	32.5 (13-82)	NA
**Clinical impression**			
Not seemingly ill	34 (14)	25 (16)	9 (11)
Moderately ill	182 (77)	123 (78)	59 (75)
Severely ill	21 (9)	10 (6)	11 (14)
**Respiratory signs and symptoms**			
Cough	174 (73)	123 (77)	51 (65)
Dyspnea	142 (60)	100 (63)	42 (53)
Abnormal lung sounds	151 (63)	98 (62)	53 (67)
If yes, unilateral sounds	79 (52)	50 (52)	29 (58)
Tachypnea	69 (29)	45 (28)	24 (30)
If yes, respiration rate, mean (SD)	29.2 (6.0)	29.8 (6.2)	28.1 (5.7)
Oxygen saturation, mean (SD)	89.6 (6.3)	91.2 (5.1)	86.8 (7.7)
**Systemic signs and symptoms**			
Hypotension	5 (2)	2 (1)	3 (4)
Tachycardia	52 (22)	21 (13)	31 (39)
Fever (≥38°C)	72 (33)	45 (29)	27 (40)
Delirium	11 (5)	4 (3)	7 (9)

IQR=interquartile range; NA=not applicable; SD=standard deviation.

* Within group valid percentages are shown. For all variables, missing data (missing from system) were <3%, with exception of respiration rate (7.9%) and fever (8.3%).

Antibiotics were prescribed at initial consultation for 84 patients (53.5%) in the intervention group and 65 patients (82.3%) in the control group. The between group difference in initial antibiotic prescribing at any point from initial consultation to follow-up was 23.6% (table S2). The naïve logistic regression analysis (table 2) showed that patients in the intervention group had 4.04 higher odds (95% confidence interval 2.09 to 7.78) of not being prescribed antibiotics at initial consultation compared with those in the control group. In the adjusted model and in the final model, this effect was retained with increased magnitude: compared with patients in the control group, those in the intervention group had 4.93 higher odds (1.91 to 12.73) of not being prescribed antibiotics at initial consultation when controlling for treating physician and baseline characteristics. The between group difference for patients with chronic obstructive pulmonary disease was 34.9%; that is, 20/45 (44.4%) of patients with chronic obstructive pulmonary disease in the intervention group received antibiotics at initial consultation compared with 23/29 (79.3%) in the control group.

**Table 2:** Effect of C reactive protein point-of-care testing on primary and secondary outcome measures. Data are numbers (percentages) unless stated otherwise

Outcome measure	Intervention group	Control group	OR (95% CI)	P value
**Primary outcome measure**				
No antibiotic prescription at initial consultation	73 (46.5)	14 (17.7)	Naïve model: 4.04 (2.09 to 7.78)	<0.001*
			Adjusted model: 4.26 (1.90 to 9.54)	<0.001*
			Final model: 4.93 (1.91 to 12.73)	0.001*
**Secondary outcome measures during follow-up period**				
Full recovery† at three weeks	121 (86.4)	69 (90.8)	0.49 (0.21 to 1.12)	0.09
Use of additional diagnostics‡ at one week and three weeks	50 (16.9)	32 (20.6)	0.72 (0.38 to 1.36)	0.31
Any changes in treatment policy‡ at one week and three weeks	36 (12.2)	26 (16.8)	0.53 (0.26 to 1.08)	0.08
Hospital admission§ at baseline, one week, or three weeks	10 (7.2)	5 (6.5)	1.12 (0.37 to 3.39)	0.85
All cause mortality§ at baseline, one week, or three weeks	5 (3.5)	1 (1.3)	2.76 (0.32 to 24.04)	0.36

Percentages shown are valid percentages. Naïve model=logistic regression analysis without adjustment for clustering of patients within treating physicians and without correction for baseline characteristics (n=236). Adjusted model=with adjustment for clustering of patients within treating physicians (logistic generalised estimating equation analysis; n=236). Final model=with correction for clustering and baseline characteristics (patient n=223, physician n=81, average number of patients per physician=3, range 1-27).

Reasons for hospital admission: worsening of symptoms (n=5); not responding to antibiotic treatment (n=2); fracture (n=2); chest pain (n=1); blood transfusion needed because of anaemia (n=1); pneumonia sepsis (n=1); hyponatremia (n=1); clinical worsening because of poor drug adherence (n=1); because of the lower respiratory tract infection, CRP=86 mg/L, no further reasons mentioned (n=1). Complications in patients not admitted to hospital: decreased consciousness (n=1).

* Statistical significance at the P<0.05 level.

† Analysed using the final model structure.

‡ Analysed using the final model structure, but with correction for clustering on the patient level instead of physician, to account for the longitudinal nature of this analysis; that is, only one cluster level can be added in logistic generalised estimating equation analysis. Any changes in treatment policy relate to a new start, switching, cessation, or prolongation of an antibiotic treatment course.

§ Naïve model structure because of low occurrence of events.

**Fig 1:**
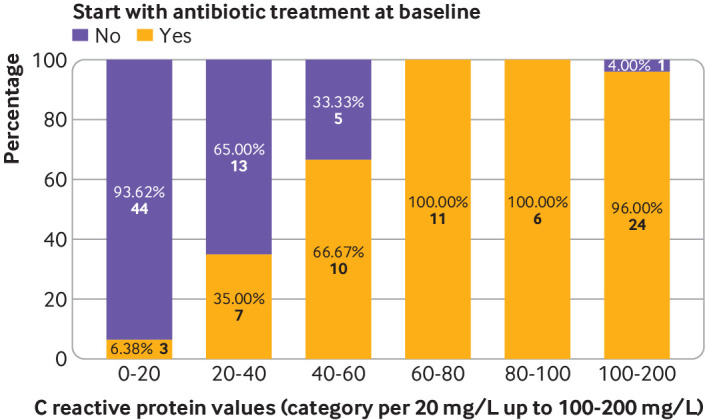
Antibiotic prescribing at initial consultation across categories of C reactive protein values (intervention group)

Antibiotic treatment changes (start, cessation, switch, or prolongation) occurred less often in the intervention group during follow-up compared with the control group (odds ratio 0.53, 95% confidence interval 0.26 to 1.08), irrespective of treating physician and baseline characteristics. Table S2 shows that the most pronounced differences in treatment changes between the groups were an initial start of treatment in the first week after initial consultation (intervention *v* control group: 6.7% *v* 2.6%) and a switch in treatment regimen (4.6% *v* 10.3% at one week and 1.4% *v* 7.8% at three weeks). Cessation or prolongation of antibiotic treatment during follow-up was uncommon (<4%). Figure 1 shows that at initial consultation, antibiotics were increasingly prescribed for patients with CRP levels ≥40 mg/L, and they were almost always prescribed when CRP level ≥60 mg/L. Figure S1 provides more detailed information, with physician specific antibiotic prescribing decisions across CRP values.

Hospital admission and mortality were uncommon in both groups, therefore we could not perform logistic generalised estimating equation analyses for these outcomes with the final model structure. Instead we performed uncorrected logistic regression analyses (table 2); however, all cause mortality findings should be interpreted with caution because of statistical imprecision. Patients in the intervention group did not have statistically significant higher odds of mortality (odds ratio 2.76, 95% confidence interval 0.32 to 24.04) and hospital admission (1.12, 0.37 to 3.39). All participants who were admitted to hospital or died received antibiotics at baseline or immediately upon hospital admission, except for one patient who was admitted to hospital and was then diagnosed with a pneumothorax. The point percentage difference in full recovery at three weeks was 4.4% between the intervention and control groups. The intervention group had lower odds (0.49, 0.21 to 1.12) of full recovery at three weeks compared with the control group, regardless of treating physician and baseline characteristics. Among the 26 patients not fully recovered in both groups, 21 received antibiotics at initial consultation (data not shown).

**Fig 2:**
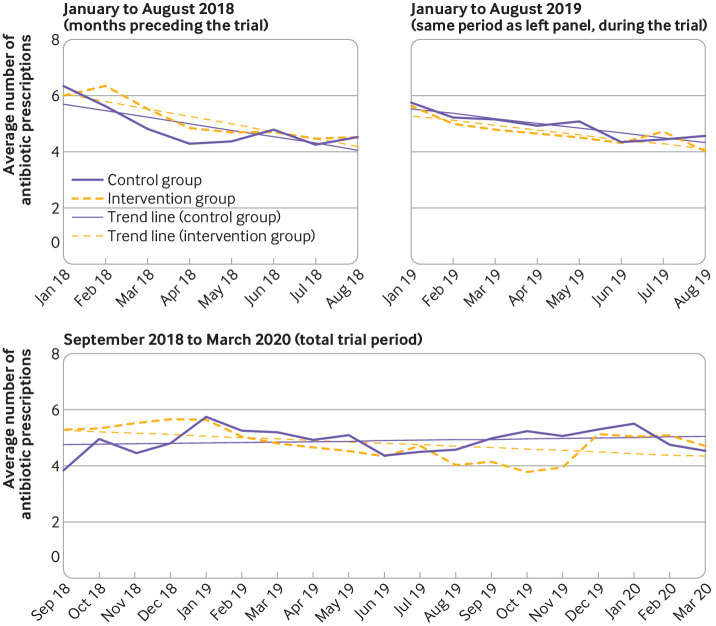
Average number of antibiotic prescriptions (all indications, ATC code J01) per 1000 resident days per month in the intervention group and control group

Figure 2 presents pharmacy dispensing data. The top left panel shows that the average number of antibiotic prescriptions per 1000 resident days per month seemed to decline in the intervention and control groups before the start of the trial. The top right panel shows that this trend was similar for the same months a year later during the trial. Overall, total antibiotic prescribing continued to decrease during the trial for the intervention group, but marginally increased in the control group (fig 2, lower panel). Figure S2 showed a less pronounced difference between the intervention and control groups during the trial for the sum of defined daily dose per 1000 resident days.

## Discussion

### Principal findings

In this cluster randomised controlled trial we showed that CRP POCT for nursing home residents with suspected lower respiratory tract infection resulted in a large, clinically relevant and safe reduction in antibiotic prescribing at initial consultation in comparison with usual care. The between group difference at initial consultation (28.8%) was larger than the anticipated 15% clinically relevant difference. During follow-up, this difference was smaller (23.6%). The low occurrence of hospital admission and mortality, and the relatively low between group difference in full recovery at three weeks indicate safe use of CRP POCT.

### Strengths and limitations of the study

A strength of the study involved the pragmatic design, which increases generalisability of the results.^18^ ^33^ ^34^ Additionally, the use of pharmacy dispensing data corroborated the findings of our trial that related to total antibiotic prescribing. The slightly increasing trend of total antibiotic prescribing in the control group during the trial also suggested that there was no profound Hawthorne effect (lower prescribing due to the awareness of being observed).^6^ ^35^ However, pharmacy dispensing data should be carefully interpreted because this analysis was exploratory and involved all residents, including those not in the trial, and comprised antibiotics for all types of infections. Another strength was the use of electronic case report forms, which improved efficiency of data collection and minimised missing data. The collected data allowed for adequate characterisation of our study population.

A limitation to the data collection was the absence of a standardised question about changes in diagnosis (all initially suspected lower respiratory tract infections were included). Therefore, we could not explore or correct for different diagnoses with regard to full recovery at three weeks. Furthermore, physicians filled out any potential complications in text fields of clinical status or reasons for hospital admission (see table 2 footnote). Complications rarely occurred, but the questionnaire would have been improved by using a predefined list of complications to ensure distinction between complications and different diagnoses, for example, heart failure.

Another potential limitation was the differential inclusion number between groups. A general concern for cluster randomised trials with inclusion post randomisation is selection bias.^34^ However, we suspect that the differential inclusion numbers between groups might have been due to increased awareness of UPCARE study participation in general among intervention group physicians because of CRP POCT availability. We observed small between group differences in severity of illness, type of ward, and presence of certain comorbid diseases. Differences in severity of illness and presence of specific comorbid diseases, such as chronic obstructive pulmonary disease, might relate to likelihood of antibiotic treatment^6^ ^14^; however, we corrected for these baseline characteristics in our analyses. Therefore, we do not assume that these between group differences impact on our conclusions, although the potential for unmeasured confounding remains.^18^

In our main analysis we corrected for clustering only at the physician level. We also checked potential clustering at the nursing home level, but the intra-cluster correlation coefficient at the nursing home level was near zero in a mixed model analysis that included both the physician and nursing home levels, while clustering did appear at the physician level (intra-cluster correlation coefficient for physician in three level model 0.175). Finally, we did not achieve the initially anticipated sample size. However, given the observed clustering and magnitude of the effect, the results seem sufficiently powered for the primary outcome measure.

### Meaning of the study

The findings echo those of large trials in general practice that have shown decreased antibiotic prescribing in CRP guided groups compared with usual care groups.^18^ ^27^ ^29^ The effect on antibiotic prescribing during the complete follow-up period was even more pronounced in the current study compared with studies in general practice.^18^ ^27^ Additionally, in one of these studies, CRP POCT had a similar effect on antibiotic prescribing across countries despite heterogeneity in setting and baseline prescribing rates.^29^ Interestingly, the antibiotic prescribing rates in both groups were lower than the estimated rates in the sample size calculation (53.5% and 82.3% *v* 80% and 95%). Potentially, this relates to generally increased attention to antimicrobial resistance, for instance in the media and with increasing developments in antibiotic stewardship efforts.^31^ A point prevalence survey in 2016-17 showed a slightly lower prevalence of antibiotic use in Dutch nursing homes compared with the mean for all participating European long term care facilities.^36^ Despite differences in case mix of long term care residents, CRP POCT might decrease antibiotic prescribing in nursing homes across countries in a comparable way.^29^ ^36^ Furthermore, among the CRP guided decisions in the intervention group, the decision for non-prescribing was frequently taken when CRP levels were between 20 and 40 mg/L, similar to findings from patients with chronic obstructive pulmonary disease in general practice.^27^ This finding might indicate trust among physicians towards non-prescribing, which extends beyond the cut-off value of 20 mg/L.

The high full recovery rate in both groups, the low occurrence of hospital admissions and mortality, and the non-significant difference in odds of these secondary outcomes between groups suggest that CRP POCT can be safely used in nursing homes. Hospital admission of patients from Dutch nursing homes is generally uncommon compared with other countries, partly because of the availability of in-house specialised elderly care physicians.^37^ ^38^ The study population mostly comprised moderately ill patients with curative treatment policies, which was expected given our exclusion criteria. In this group, mortality rates were lower compared with study populations that include patients receiving palliative care.^39^

### Policy implications and future research

Because of the pragmatic trial design, we believe that the results can be generalised to other nursing homes and other countries with similar long term care facilities. Evidence for large scale implementation in nursing homes could be reinforced if our economic evaluation shows cost effectiveness of CRP POCT compared with usual care. We also conducted a process evaluation that aimed to improve implementation knowledge and practical guidance for CRP POCT. Both studies will be published separately.

In countries where hospital admission of nursing home residents is more common, new studies could focus on the effect of CRP POCT on improved patient selection for hospital admission.^38^ In the current study, physicians seemed to conform to prescribing decisions across cut-off values as proposed by the current Dutch nursing home guidelines for lower respiratory tract infections.^32^ Recently, a study showed insufficient diagnostic accuracy of CRP and consistently low CRP (<20 mg/L) in suspected urinary tract infections in nursing home residents.^40^ This finding increases the likelihood of a lower respiratory tract infection compared with a urinary tract infection when the disease focus is unclear and CRP levels are moderately raised (20-60 mg/L). However, concurrent information on causative agents and CRP levels, perhaps as a combination test with other biomarkers, for suspected lower respiratory tract infection might be useful for improved decision making when CRP levels are moderately raised.^13^ ^41^ ^42^ Finally, the findings also suggested reduced antibiotic prescribing in patients with chronic obstructive pulmonary disease, but the group size was too small to allow for firm conclusions. A study in general practice found a reduction of antibiotics in patients with chronic obstructive pulmonary disease by using CRP levels, but this finding needs confirmation in a nursing home setting.^27^

**What is already known on this topic**

Diagnostic uncertainty about suspected lower respiratory tract infections in nursing home residents contributes to inappropriate antibiotic prescribing
C reactive protein point-of-care testing effectively reduces antibiotic prescribing for respiratory tract infections and chronic obstructive pulmonary disease in primary care

**What this study adds**

C reactive protein point-of-care testing (CRP POCT) was found to safely reduce antibiotic prescribing for suspected lower respiratory tract infections in nursing home residents compared with usual care (between group difference at initial consultation 28.8%)
CRP POCT in nursing homes might contribute to reduced antibiotic use in this setting and help to combat antibiotic resistance

## Data Availability

The datasets generated and analysed during the current study will be deposited in the repository DANS (EASY) after publication of the research results, within a maximum of nine months after the end of the study. The datasets involved will be pseudonymised and can be accessed under restrictions.

## Web extra

Extra material supplied by authors

Web appendix: Supplementary appendix

## Notes

Contributors: JWMRT, LWvB, MWvT, TJMV, CMPMH, RMH, and TMB contributed to the statistical analysis plan. TMB performed the statistical analyses and drafted the manuscript. LWvB, CMPMH, TJMV, RMH, MWvT, and JWMRT contributed to the final version of the manuscript. All authors have read and approved the manuscript. TMB and LWvB accept responsibility for the overall content as guarantors. The corresponding author attests that all listed authors meet authorship criteria and that no others meeting the criteria have been omitted.

The lead authors (the manuscript’s guarantors) affirm that the manuscript is an honest, accurate, and transparent account of the study being reported; that no important aspects of the study have been omitted; and that any discrepancies from the study as planned (and, if relevant, registered) have been explained.

Dissemination to participants and related patient and public communities: There are no plans to disseminate the results of the research to study participants or the relevant patient community. We plan to disseminate the results to organisations and associations of elderly care physicians and nurses.

Provenance and peer review: Provenance and peer review: Not commissioned; externally peer reviewed.
