# Supplementary material for: Effect of C reactive protein point-of-care testing on antibiotic prescribing for lower respiratory tract infections in nursing home residents: cluster randomised controlled trial
Source: BMJ. 2021 Sep 21;374:n2198. doi: 10.1136/bmj.n2198 (PMC8453309; doi:10.1136/bmj.n2198)
Supplement: Supplementary file 1 — Web appendix: Supplementary appendix [file boet065375.ww.pdf]

Effect of C reactive protein point-of-care testing on antibiotic prescribing for lower respiratory tract infections in nursing homes residents: cluster randomised controlled trial.

Supplementary Appendix

Table of contents

Supplementary tables

Table S1. Patient status and management. P2

Table S2. Antibiotic treatment at initial consultation and changes (start, switch, cessation and prolongation) during follow-up. P3

Table S3. Type of additional diagnostics performed at initial consultation. P4

Supplementary figures

Figure S1: Physician-specific antibiotic prescribing across CRP-values P5

Figure S2: sum of Defined Daily Dose of antibiotic prescriptions (all indications, ATC-codes J01) per 1000 resident days per month in the intervention and control group. P6

Figure S3: CONSORT flow diagram. P7

Figure S4: detailed flow diagram of patient enrollment. P8

Supplementary data

Sampling quality P9

Supplementary tables

Table S1. Patient status and management

	Intervention group n (%)		Control group n (%)	
**Initial consultation**	**baseline**		**baseline**	
*Additional diagnostics performed**	29 (18)		28 (35)	
Hospitalization	1 (1)		0	
**Follow-up after 1 and 3 weeks**	**1 week**	**3 weeks**	**1 week**	**3 weeks**
Fully recovered	94 (64)	121 (86)	55 (71)	69 (91)
*Not fully recovered^*	54 (37)	19 (12)	22 (29)	7 (9)
Improved clinical status	34 (71)	5 (39)	14 (70)	1 (20)
No change in clinical status	8 (17)	3 (23)	2 (10)	2 (40)
Worsened clinical status	6 (13)	5 (39)	4 (20)	2 (40)
Discharge from nursing home	1 (1)	2 (1)	1 (1)	3 (4)
All cause mortality	3 (2)	2 (1)	0 (0)	1 (1)
Hospitalization	3 (2)	6 (4)	4 (5)	1 (1)
Discharge from hospital to nursing home between 0-1 week resp. 1-3 weeks&	3 (75)	7 (100)	2 (50)	3 (100)
*Additional diagnostics performed**	20 (13)	21 (15)	22 (28)	11 (14)

***:** *In the intervention group: excluding CRP POCT.* **^**: *Include deceased or not fully recovered patients. Percentages shown for changes in clinical status involve alive, not-fully recovered patients.* **&**: *the percentage shown reflects the proportion discharged among those hospitalized at that point during the follow-up period.*

Table S2. Antibiotic treatment at initial consultation and changes (i.e., start, switch, cessation and prolongation) during follow-up.

	Intervention group n (%)				Control group n (%)			
**Antibiotic treatment**	**Baseline**	**1w**	**3w**	**Total cases**	**Baseline**	**1w**	**3w**	**Total cases**
*Start^*	84 (53.5)	11 (7.3)	5 (3.5)	N/A	65 (82.3)	3 (3.8)	3 (3.9)	N/A
Initial start&	84 (53.5)	10 (6.7)	1 (0.6)	95 (62.5)	65 (82.3)	2 (2.6)	1 (1.3)	68 (86.1)
Cessation	N/A	1 (0.7)	1 (0.7)	2 (1.4)	N/A	1 (1.3)	0	1 (1.3)
Prolongation	N/A	4 (2.6)	3 (2.1)	7 (4.9)	N/A	3 (3.8)	1 (1.3)	3 (3.9)
Switch	N/A	7 (4.6)	2 (1.4)	9 (6.3)	N/A	8 (10.3)	6 (7.8)	12 (15.6)

***** *Reasons for switching (multiple reasons possible) were insufficient effect (N=14), allergy (N=1) and other reasons (N=9). There were no reports of side-effects or antibiotic resistance.* **^** *including patients who received antibiotic treatment at a previous time point.* **&** *excluding patients who received antibiotic treatment at a previous time point. Data of patients with a later initial start of antibiotics suggested that the reason for a later start related to worsening of symptoms (N=5, intervention group; N=1, control group), worsening of symptoms and/or increased CRP-value (N=2, intervention group; N=1, control group), treatment policy changed from palliative to active because of clinical improvement (N=1, intervention group), and unknown reasons (N=3, intervention group, N=1, control group).*

Table S3. Type of additional diagnostics performed at initial consultation

Diagnostics	Intervention group n (%)	Control group n (%)
CRP POCT	139 (87.4)	N/A (0)
CRP laboratory test	25 (15.7)	19 (24.1)
Chest X-ray (hospital)	1 (0.6)	0
Chest X-ray (nursing home)	0	0
Sputum culture	2 (1.3)	3 (3.8)
- Other	2 (1.3)	10 (12.7)

Supplementary figures

Figure S1: Physician-specific antibiotic prescribing across CRP-values.

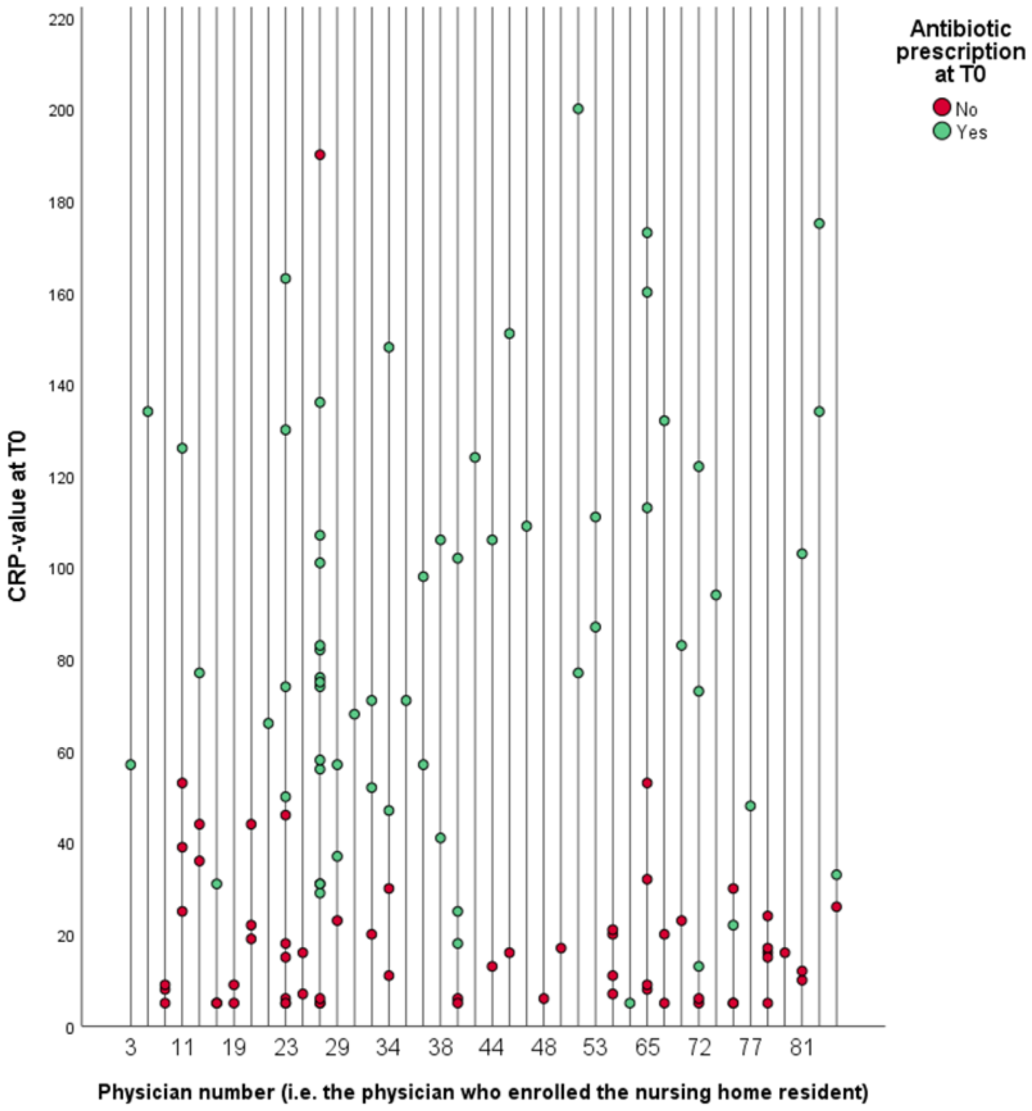

Figure S2: sum of Defined Daily Dose of antibiotic prescriptions (all indications, ATC-codes J01) per 1000 resident days per month in the intervention group and control group.

A: January until August 2018 (months preceding the trial); **B** January until August 2019 (same period as A, during the trial); **C** September 2018 until March 2020 (total trial period).

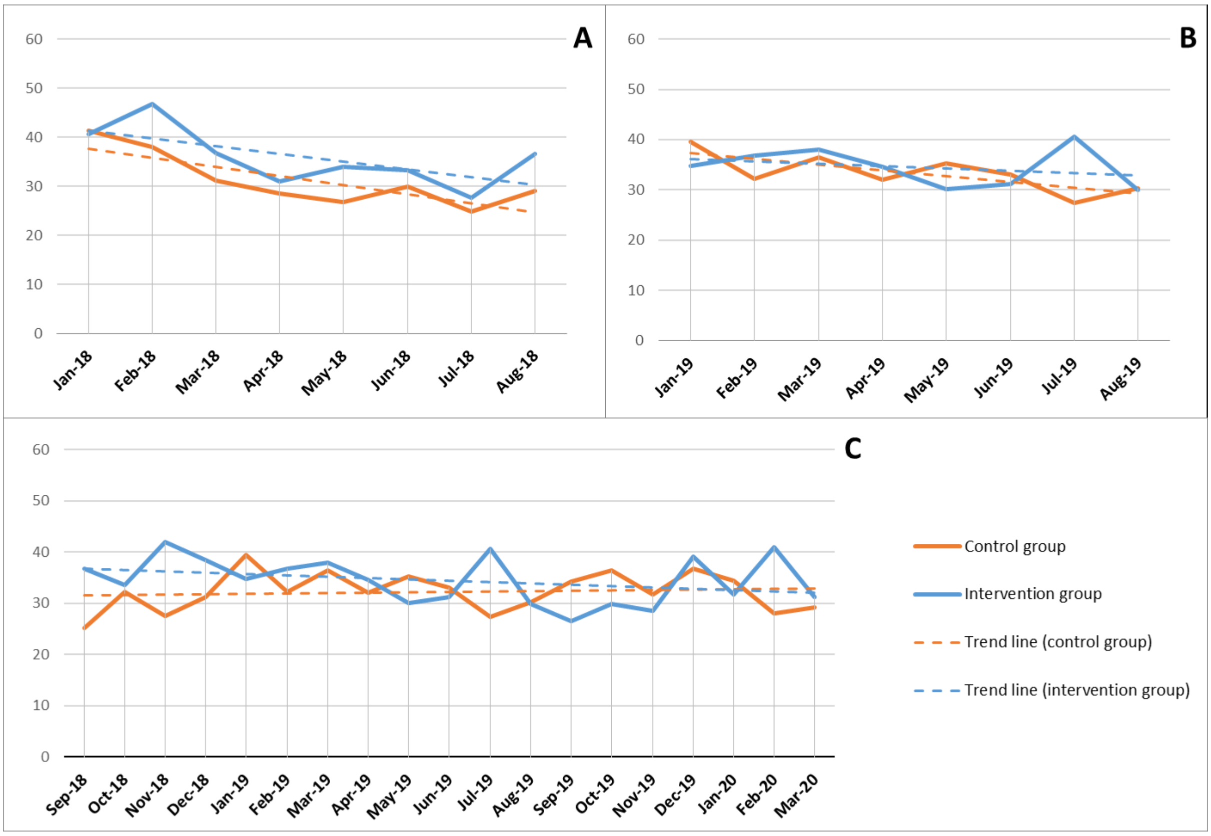

CONSORT
TRANSPARENT REPORTING of TRIALS

Figure S3. CONSORT Flow Diagram

CONSORT 2010 Flow Diagram

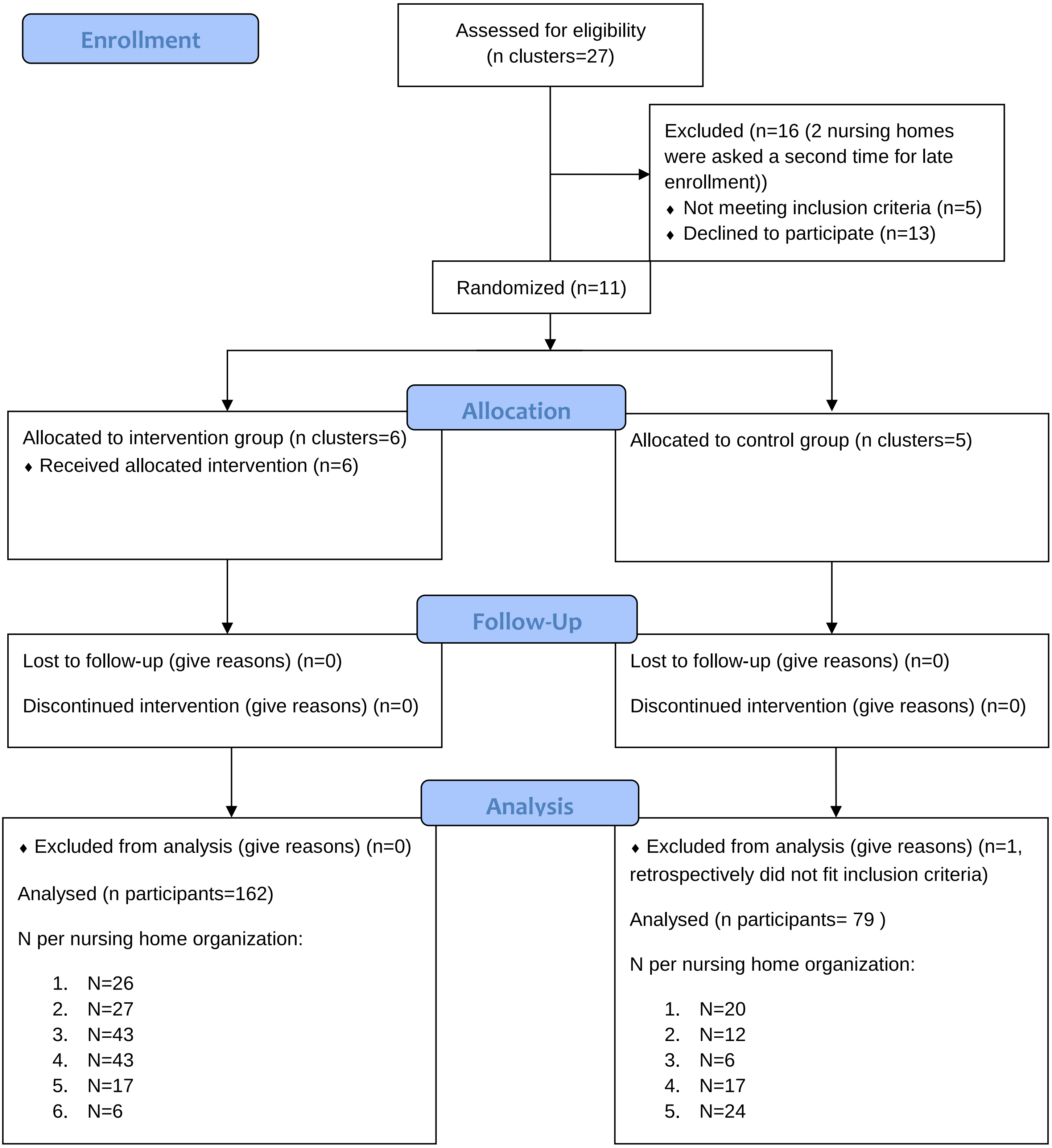

Figure S4. Detailed flow diagram of patient enrollment

Before trial start

Randomization

Ward capacity

	Intervention group Average N or % (range)	Control group Average N or % (range)
**Total beds**	361 (250-480)	418 (289-650)
**Psychogeriatric**	50% (28-74%)	47% (24-68%)
**Somatic**	29% (0-51%)	42% (21-78%)
**Geriatric rehabilitation**	19% (0-32%)	10% (0-18%)
**Other**	2% (0-7%)	2% (0-5%)

Before trial or with each new admission

All NH residents invited for participation

Average % of residents who opted-out per organization:
Control group: 11% (7-15%)
Intervention group: 7% (4-11%)

Average total number of residents to be potentially included per organization:
Control group: 372 (247-573)
Intervention group: 334 (231-456)

Number of potential participants (suspected LRTI) unknown

% refusal to consent at index consultation unknown.

Average exclusion rate (of estimated incident cases) per organization:
Control group: 7% (1-10%)
Intervention group: 5% (1-13%)

During study

Number of residents included:
Control group: 79
Intervention group: 162

Participants

	Intervention group (unknown ward: 4)	Control group
**Psychogeriatric**	55 (35%)	23 (28%)
**Somatic**	71 (45%)	42 (53%)
**Geriatric rehabilitation**	29 (18%)	11 (14%)
**Short-term residential care**	3 (2%)	3 (4%)

Supplementary data

Sampling quality

Methods

This supplementary data regarding sampling quality was assessed in a process evaluation, alongside assessment of intervention quality and implementation knowledge of CRP POCT in the UPCARE trial. Data sources that were used included: logs of communication, nursing home intake forms (i.e. information on location and ward size), anonymous overview provided by the software provider of the case report forms (i.e. the number of patients opting out and excluded per organization), trial data (i.e. illness severity and inclusion moment), and a process evaluation questionnaire (i.e. information on enrollment). The main focus of the questionnaire was intervention quality, therefore, the questionnaire was only sent to physicians and nurses in the intervention group.

Results

Recruitment and randomization

Figure S3 shows that the research team invited 27 NH organizations, of which ten agreed to participate at the initial invitation. One NH agreed at a later time and started April 2019. Reasons for not participating were: not fitting inclusion criteria (five), organizational issues (seven), no time because of participation in another study (three), misfit with local research priorities (one), negative experience with CRP POCT in the organization (one), not willing to take the risk of being randomized to the control group (one). Participating NHs were located in different geographical regions in the Netherlands. Figure S4 shows, amongst others, the average ward sizes of the intervention and control group after randomization. The randomization resulted in similar distribution of psychogeriatric ward size per organization between the intervention and control group. The intervention group organizations had on average fewer somatic residents and more geriatric rehabilitation patients.

Informed consent, participation rate and reach

Figure S4 also shows the flow chart of inclusion, with opt-out and exclusion rates, and ward capacity. After receiving the information letter, 7% intervention group and 11% control group NH residents or their legal representatives chose to opt-out. Of eligible participants, 5% (intervention group) and 7% (control group) were excluded because of our exclusion criteria, which was lower than the anticipated 10%. The final number of participants was 79 in the control group and 162 in the intervention group.

The between-group difference in ward type of participants ranged between 2-8% for the different types of wards. For instance, the proportion of psychogeriatric residents among all participants in the intervention group was 35% compared to 29% in the control group. Most participants were moderately ill in both groups (78% resp. 75%). The majority of physicians who included participants indicated in the questionnaire (response rate: 33%) that they included 25-50% of potential cases. Reasons that were mentioned for being unable to include patients were difficulty in obtaining informed consent, a high workload, and high turnover rates at geriatric rehabilitation wards. Participant enrollment most often took place during office hours (87%, range 63-100%), 9% (0-29%) during weekends, and 5% (0-12%) during nights.
